# Interaction of Water-Soluble CdTe Quantum Dots with Bovine Serum Albumin

**DOI:** 10.1007/s11671-010-9740-9

**Published:** 2010-08-22

**Authors:** Vilius Poderys, Marija Matulionyte, Algirdas Selskis, Ricardas Rotomskis

**Affiliations:** 1 Laboratory of Biomedical Physics, Vilnius University Institute of Oncology, Vilnius, Lithuania; 2 Biophotonics Laboratory, Quantum Electronics Department, Physics Faculty, Vilnius University, Vilnius, Lithuania; 3 Department of Material Structure, Institute of Chemistry, Vilnius, Lithuania

**Keywords:** Quantum dots, Aggregation, Bovine serum albumin, Interaction, Stabilization

## Abstract

Semiconductor nanoparticles (quantum dots) are promising fluorescent markers, but it is very little known about interaction of quantum dots with biological molecules. In this study, interaction of CdTe quantum dots coated with thioglycolic acid (TGA) with bovine serum albumin was investigated. Steady state spectroscopy, atomic force microscopy, electron microscopy and dynamic light scattering methods were used. It was explored how bovine serum albumin affects stability and spectral properties of quantum dots in aqueous media. CdTe–TGA quantum dots in aqueous solution appeared to be not stable and precipitated. Interaction with bovine serum albumin significantly enhanced stability and photoluminescence quantum yield of quantum dots and prevented quantum dots from aggregating.

## Introduction

Since the first time fluorescent semiconductor nanoparticles (quantum dots) were synthesized, they are widely explored due to their possible applications in many fields, including medicine. Tunable emission wavelength, broad absorption and sharp emission spectra, high quantum yield (QY), resistance to chemical degradation and photo bleaching and versatility in surface modification make quantum dots very promising fluorescent markers [1].

Quantum dots can be used for live cell labeling ex vivo, detection and imaging of cancer cells ex vivo [2], as a specific marker for healthy and diseased tissues labeling [3], for labeling healthy and cancerous cells in vivo [4] and for treatment of cancer using photodynamic therapy [5]. Despite all unique photo physical properties, some problems must be solved before quantum dots can be successfully applied in medicine. Quantum dots usually are water insoluble and made of materials that are toxic for biological objects (Cd, Se). To make them suitable for application in medicine, surface of quantum dots has to be modified to make them water-soluble and resistant to biological media. After injection of quantum dots to live organisms, they are exposed to various biomolecules (ions, proteins, blood cells, etc.). This could lead to degradation of quantum dot coating or quantum dot itself. In this case, toxic Cd^2+^ ions are released and can cause damage to cells or even cell death.

A lot of research is done to better understand quantum dots synthesis [6] growth [7] and modification [1]. Recently, the interaction of quantum dots with biomolecules attracted much interest and is studied using various methods, such as atomic force microscopy, gel electrophoresis, dynamic light scattering, size-exclusion high-performance liquid chromatography, circular dichroism spectroscopy and fluorescence correlation spectroscopy [7-11]. It was shown that interaction of quantum dots with biological molecules can enhance optical properties and stability of quantum dots [12-14] or it may oppositely lead to their degradation [15]. Serum albumin is one of the most studied proteins. It is the most abundant protein in blood plasma and plays a key role in the transport of a large number of metabolites, endogenous ligands, fatty acids, bilirubin, hormones, anesthetics and other commonly used drugs.

In this study, we investigated effect of interaction between bovine serum albumin (BSA) and water-soluble CdTe quantum dots in aqueous solutions using microscopy and spectroscopy methods.

## Materials and Methods

Quantum dots solutions were prepared by dissolving CdTe quantum dots coated with thioglycolic acid (*λ*_PL_ = 550 ± 5 nm, PlasmaChem GmbH, Germany) in deionized water (pH≈6) or saline (0.9% NaCl solution, pH≈5.6). Experiments of CdTe quantum dots solution with protein were performed by adding a small amount of concentrated bovine serum albumin (BSA) (BSA, V fraction, *M* = 69,000 g/mol, Sigma, Germany) solution in saline to the quantum dots solution.

Spectral measurements were performed immediately after preparation of solutions. Absorbance spectra were measured with Varian Cary Win UV (Varian Inc., Australia) absorption spectrometer. Photoluminescence spectra were measured with Varian Cary Eclipse (Varian Inc., Australia) and PerkinElmer LS 50B (PerkinElmer, USA) fluorimeters. Photoluminescence excitation wavelength was 405 nm, excitation slits were 5 nm and emission slits 5 and 4 nm for Varian Cary Eclipse and PerkinElmer LS 50B, respectively. Measurements were taken in 1-cm path length quartz cells (Hellma, Germany). Samples for atomic force microscopy measurements were prepared by casting a drop (40 μl) of solution on freshly cleaved V-1 grade muscovite mica (SPI supplies, USA) spinning at 1,000 rpm. Atomic force microscope (AFM) diInnova (Veeco instruments inc., USA) was used to take 3-dimensional (3-D) images of quantum dots. Measurements were performed in tapping mode in air; RTESP7 cantilevers (Veeco instruments inc., USA) were used. Samples for scanning transmission electron microscopy (STEM) measurements were prepared by casting a drop of solution on TEM grid and drying it in ambient air. STEM images were obtained with HITACHI SU8000 microscope (Hitachi High-Technologies Corporation, Japan). Malvern Zetasizer Nano S (Malvern Instruments Ltd., England) was used to determine particles size distributions in investigated solutions.

## Results

**Figure 1:**
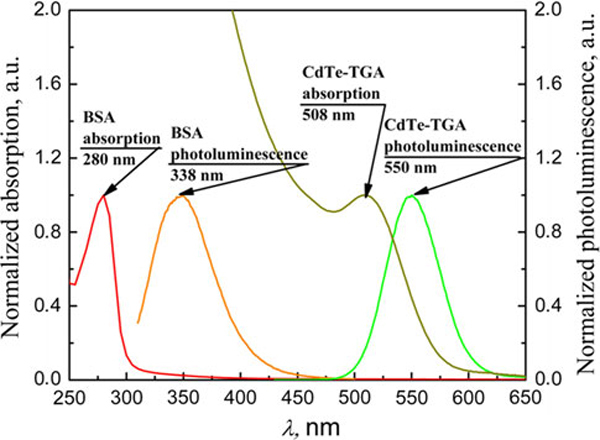
**Normalized photoluminescence and normalized absorption spectra of bovine serum albumin (BSA) and CdTe quantum dots coated with thioglycolic acid**.

**Figure 2:**
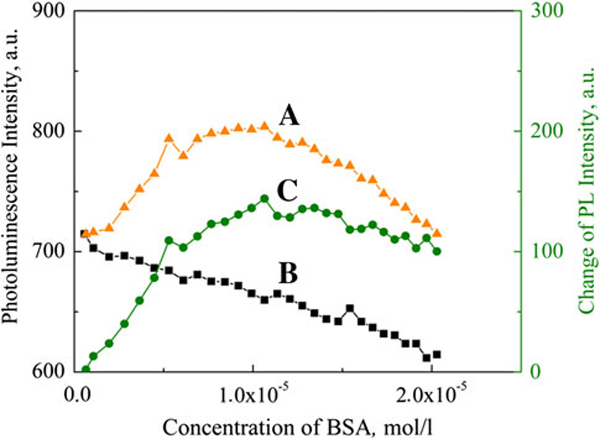
**CdTe quantum dots (CdTe *c* = 7.5 × 10^-6^ mol/l) photoluminescence intensity (at 550 nm): *A* during titration with BSA (*c* = 10^-4^ mol/l), *B* titrating with saline, *C* change of photoluminescence intensity caused by BSA (dilution effect is eliminated)**.

Normalized photoluminescence and absorption spectra of BSA and CdTe quantum dots coated with thioglycolic acid is presented in Figure 1. BSA has absorption band in UV region at 280 nm, and fluorescence band peak is at 338 nm. CdTe–TGA quantum dots absorb light in wide spectral region and have excitonic absorption band at 508 nm, and photoluminescence band peak of quantum dots solution is at 550 nm. Titration of freshly prepared quantum dots solution with BSA showed that addition of protein to CdTe quantum dots solution increases photoluminescence intensity of quantum dots (simultaneously a slight (~4 nm) bathochromic shift of quantum dots excitonic absorption band is observed). This effect was observed until 10^-5^ mol/l BSA concentration was reached. Further increase of BSA concentration in quantum dots solution induced slight decrease in photoluminescence intensity (Figure 2, curve A). Constant decrease in CdTe quantum dots solution photoluminescence intensity was observed, when CdTe quantum dots solution was titrated with saline (Figure 2, curve B). This constant decrease in photoluminescence intensity was caused by decreasing concentration of quantum dots (dilution effect). Curve C (Figure 2) shows CdTe quantum dots photoluminescence intensity change caused by CdTe–BSA interaction (dilution effect is eliminated). The biggest increase in CdTe quantum dots photoluminescence intensity (120% of initial value) was observed when ratio of BSA/quantum dot was 1.75:1.

**Figure 3:**
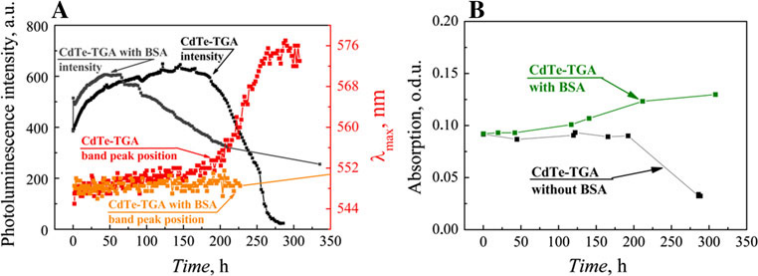
**a dynamics of CdTe quantum dots solution (*c* = 6 × 10^-6^ mol/l) photoluminescence intensity (measured at peak position) and photoluminescence band peak position, b absorption (at excitonic absorption band maximum) dynamics of CdTe quantum dots**.

Dynamics of quantum dots photoluminescence properties (photoluminescence intensity and photoluminescence band peak position) in solutions with BSA and without BSA are presented in Figure 3. Photoluminescence intensity of CdTe–TGA quantum dots solution (*c* = 6 × 10^-6^ mol/l) without bovine serum albumin was increasing for the first 144 h (Figure 3, curve A). Photoluminescence band maximum position and width stayed intact. After 144-h photoluminescence intensity started to decrease, band started to narrow and shift to longer wavelength region. Simultaneously absorption slightly decreased (Figure 3, curve B). Decrease in quantum dots photoluminescence intensity and bathochromic shift of photoluminescence band indicates aggregation of quantum dots. After 9 days, precipitate of large aggregates appeared in quantum dots solution.

A sudden increase in photoluminescence intensity (by 27%) was observed after protein was added to the CdTe quantum dots solution in saline (Figure 3a). Photoluminescence intensity further increased for approximately 40 h. Later photoluminescence intensity started decreasing, but decrease in intensity was quite slow and at longer time scale became negligible (even after 6 months no precipitate was observed). Photoluminescence band width and maximum position remained constant, and absorption intensity slightly increased. This indicates that core of quantum dot remained intact.

**Figure 4:**
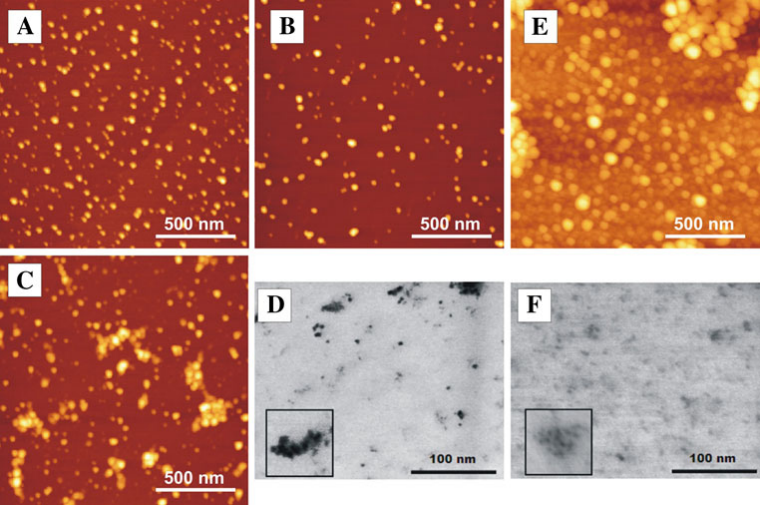
**AFM (a, b, c, e) and STEM (d, f) images of CdTe quantum dots**. **a**–**c** AFM images of quantum dots dispersed on mica (dispersed from aqueous solution kept for: **a** 40 min, **b** 5 h, **c** 24 h), **d** STEM image of quantum dots dispersed on TEM grid (dispersed from solution kept for 48 h), **e** AFM image of quantum dots with BSA dispersed on mica (dispersed from aqueous solution kept for 2 months), **f** STEM image of quantum dots with BSA (dispersed from aqueous solution kept for 2 months). Inserts (in **d** and **f** images) show magnified view (40 nm × 40 nm). Concentrations of solutions used for sample preparation were 6 × 10^-6^ mol/l.

Investigation of quantum dot size with atomic force microscope (AFM) and scanning electron transmission microscope (STEM) showed that in solution without protein quantum dots aggregate (Figure 4a–d). AFM image of quantum dots, deposited from solution that was kept for 40 min, is presented in Figure 4a. A lot of small round structures were present on the surface. These structures were ~2.5 nm in height and ~25 nm in width. Shape of colloidal quantum dots should be close to spherical (width of quantum dot should be approximately equal to height). Height of these structures is approximately equal to a height of single quantum dot, but width was much bigger. This could be explained by AFM imaging artifact called "tip imaging". It is also possible that these small structures are not single quantum dots but few quantum dots attached to each other. AFM image of quantum dots deposited from solution that was kept for 5 h shows larger structures (Figure 4b). Height and width of these structures varied in broader range. Some small structures (height–2.5 nm, width–20 nm) could be seen, but bigger structures (up to 9 nm in height and up to 70 nm in width) were also present. Image of sample prepared from solution that was kept for 24 h (Figure 4c) showed that sizes of the structures increased even more (height–up to 13 nm, width–up to 150 nm). In STEM images (Figure 4d), obtained 2 days after solution preparation, various size structures (much larger than single quantum dots) were seen. This shows that CdTe–TGA quantum dots dissolved in aqueous solution are not stable, and aggregates and forms large clusters of quantum dots.

AFM image (Figure 4e) of sample prepared from CdTe quantum dots solution in saline with BSA (solution was kept for 2 months) showed that there were no large structures that could form precipitate, but there were plenty of round structures that were 9–20 nm in height and 40–60 nm in width. Height of structures seen in image (9–20 nm) was bigger than height of single quantum dot (~2.5 nm). BSA is heart-shaped molecule; its approximate size is 8 nm × 8 nm × 3 nm [14]. Structures observed in AFM image were a bit bigger than BSA molecules. Structures observed in AFM image could be CdTe quantum dots coated with BSA. In STEM image, only small structures (single quantum dot) ~3 nm in diameter are seen. In bigger collections, quantum dots are separated one from another by ~3 nm (Figure 4f). Interaction of quantum dots with BSA could lead to the formation of additional quantum dot coating layer that prevents quantum dots from aggregation. Additional coating layer is not visible in STEM image because BSA is formed of light atoms that are not visible in STEM images.

**Figure 5:**
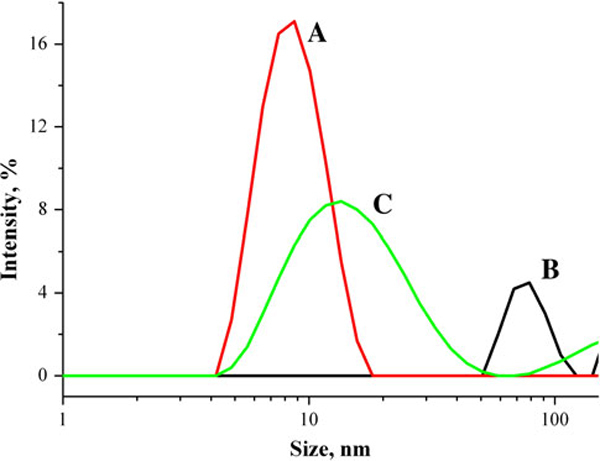
**Particle size distributions: *A* in aqueous BSA solution (c = 10^-5^ mol/l), *B* in aqueous CdTe–TGA quantum dots solution (*c* = 6 × 10^-6^ mol/l), *C* in aqueous CdTe–TGA quantum dots solution (*c* = 6 × 10^-6^ mol/l) with BSA (*c* = 10^-5^ mol/l)**. All solutions were kept for 1 week.

Particle size distributions in BSA solution, CdTe–TGA quantum dots solution and CdTe–TGA quantum dots solution with BSA are presented in Figure 5 (solutions were kept for 1 week). Average diameter of particles in BSA solution is 8.7 nm. This result very well coincides with dimensions of BSA molecule presented in literature [16]. Sizes of particles present in CdTe quantum dots solution are bigger than 50 nm in diameter, much bigger than size of single quantum dot (that should be approximately 2–3 nm). This shows that quantum dots formed aggregates and confirms results obtained with AFM and STEM. Particle size distribution in CdTe–TGA with BSA solution shows that in this solution average particle size is slightly bigger (diameter ~12.5 nm) than in BSA solution (diameter ~8.7 nm). This shows that CdTe–TGA quantum dots interact with BSA and form quantum dot–protein complex whose size is approximately 12.5 nm.

## Discussion

**Figure 6:**
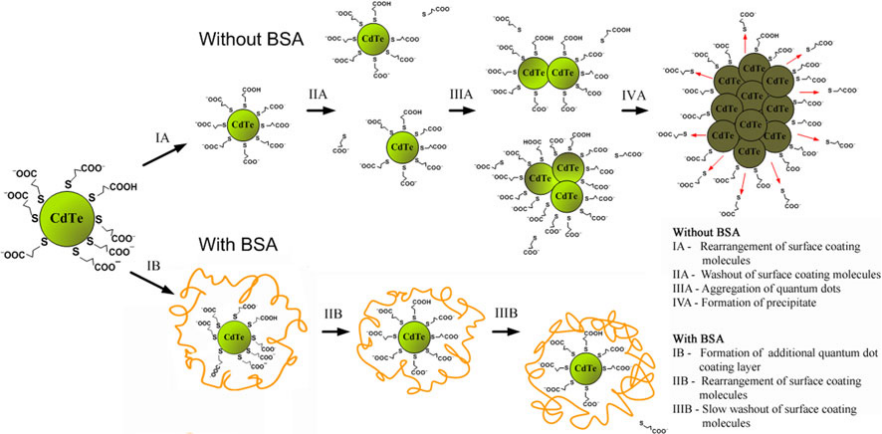
**Model of CdTe–TGA aggregation and interaction with bovine serum albumin**.

Our proposed model explaining spectral dynamics of CdTe–TGA quantum dots in aqueous solution with and without BSA is presented in Figure 6.

Dynamics of photoluminescence properties of investigated solutions (presented in Figure 3) show two phases—growth of photoluminescence and decrease of photoluminescence. In the first phase, photoluminescence of quantum dots increased in both investigated solutions (quantum dots without protein and quantum dots with protein). Despite quite large increase in photoluminescence spectra, changes in absorption spectrum were very small. During this phase, photoluminescence band peak position and photoluminescence band width remained constant. These changes indicate that core of quantum dot remains intact. Core degradation would cause blue shift of photoluminescence band; aggregation of quantum dots would cause a red shift. Change in photoluminescence intensity indicates that properties of quantum dot coating (or coating itself) are changing: molecules coating core of quantum dot are rearranging, being replaced by other molecules of being washed-out. Theoretically, increase in quantum dots photoluminescence intensity is explained by decrease in non-radiative transitions or their speeds. Decrease in defects on quantum dots surface would cause this effect [17]. Another process that can change intensity of quantum dots photoluminescence is aggregation. Aggregation of quantum dots decreases photoluminescence quantum yield. Slow dissolution (monomerization) of quantum dots powder (aggregates) could cause increasing photoluminescence intensity due to increased photoluminescence quantum yield of single quantum dots compared with aggregated form. More detailed investigation into absorption spectrum dynamics during first day after preparation of solution contradicts to this explanation. Absorption of quantum dots dissolved in deionized water decreases during first day. This decrease can be explained by aggregation of quantum dots. Aggregation of quantum dots leads to decrease in absorption intensity, red shift, broadening and photoluminescence band intensity decrease. But in first phase, width and wavelength of photoluminescence band do not change, whereas photoluminescence intensity increases. So these changes are caused not by aggregation of quantum dots but by changes in quantum dot coating. CdTe–TGA quantum dots are fluorescent nanoparticles composed of CdTe core and TGA coating. Rearrangement of quantum dot coating can lead to decrease in defects on quantum dot surface and increase in photoluminescence quantum yield. Sudden increase in quantum dots photoluminescence band intensity, after adding BSA to solution, shows that interaction of quantum dots with BSA strongly increases photoluminescence quantum yield. Photoluminescence decay measurements presented in literature [18] confirm this result. Photoluminescence decay of quantum dots with BSA is tri-exponential, while photoluminescence decay of quantum dots is described with four exponents. This shows that addition of protein eliminates one excitation relaxation path. Photoluminescence lifetime analysis shows that fastest relaxation component (τ_1_ = 3.4 ns) disappears [18]. Fastest relaxation component is caused by defects of quantum dots [19]. Elimination of this component leads to increase in quantum dots photoluminescence quantum yield. So increase in photoluminescence intensity at the first phase is caused by rearrangement of TGA molecules (Figure 6IA, IB).

In the second phase, photoluminescence of quantum dots starts to decrease. TGA molecules are not covalently bound to CdTe core (they are attached to it by coordinating bonds [20]) and probably are washing out slowly (Figure 6IIA, IIIB). This process increases number of defects on quantum dots surface and leads to decrease in photoluminescence quantum yield. AFM and STEM images (Figure 4a–d) show that quantum dots in aqueous media aggregate. TGA coating makes CdTe quantum dots water soluble. Washing out of coating decreases water solubility of quantum dots, increases aggregation speed (Figure 6IIIA) and leads to formation of precipitate (Figure 6IVA). In the second phase, effects of aggregation (decrease in photoluminescence intensity and red shift of photoluminescence band) are seen in quantum dots solution without protein (Figure 3a).

Second phase is different for quantum dots solution with protein. In this case, photoluminescence decreases slowly and after some time stabilizes. Position of photoluminescence band does not change during this phase. This shows that quantum dots in the presence of protein do not aggregate, and protein prevents the degradation of quantum dot coating and aggregation of quantum dots.

## Conclusions

This study showed that water-soluble CdTe–TGA quantum dots in aqueous solutions are not stable. Spectroscopic and atomic force microscopy measurements showed that quantum dots aggregate in solution, and 9 days after preparation of solution, precipitate was observed. BSA interacts with CdTe–TGA quantum dots, prevents them from aggregating, increases photoluminescence quantum yield and makes them stable. This effect is achieved by forming a new layer of quantum dot coating.
